# Not for children’s ears? Parents’ insights into early childhood overweight and obesity treatment

**DOI:** 10.1080/02813432.2025.2531958

**Published:** 2025-07-15

**Authors:** My Sjunnestrand, Nicklas Neuman, Anna Ek, Karin Nordin, Ximena Ramos Salas, Kajsa Järvholm, Karin Eli, Paulina Nowicka

**Affiliations:** ^a^ Department of Food Studies, Nutrition and Dietetics, Uppsala University, Uppsala, Sweden; ^b^ Department of Clinical Science, Intervention and Technology (CLINTEC), Stockholm, Sweden; ^c^ Replica Communications, Kristianstad, Sweden and Bias 180, Dundas, Canada; ^d^ Department of Psychology, Lund University, Lund, Sweden; ^e^ Warwick Medical School, University of Warwick, Coventry, UK

**Keywords:** Childhood obesity, childhood obesity treatment, healthcare interactions, weight-related communication, healthcare, stigma

## Abstract

This study offers novel insights into parents’ experiences of healthcare interactions during weight-related visits. Data were collected through the More and Less Europe study, a randomized controlled trial evaluating a parent support program for children (aged 2–6 years) with overweight or obesity in Sweden, Romania, and Spain to capture parents’ experiences of healthcare interactions. Semi-structured interviews were conducted with 45 Swedish parents (71% mothers, 60% with university degree, 51% of migrant background) of 45 children (mean age 7.1 years, SD 1.3, 76% girls) who received standard treatment for overweight or obesity. The interviews were analyzed thematically, identifying two themes: (1) *Support or blame,* with subthemes *Validating family-centeredness, Overly generic advice,* and *Stigma and the sense of failing;* (2) *The place and role of the child,* with subthemes *Neutral, honest, and direct communication, Not for children’s ears*, and *Framing the message with care.* While some parents described supportive interactions, others expressed disappointment with generic advice and inadequate support. Some recalled stigmatization, sometimes in the child’s presence, raising concerns about the child’s well-being. Approaches to preparing children for visits ranged from neutral to direct explanations. Parents expressed contrasting views on children’s presence during weight-related discussions: while some felt such discussions would harm their child, others supported children’s presence in open and age-appropriate discussion. This study fills a critical gap in obesity management communication by highlighting parental concerns about children’s exposure to weight-related discussions. Addressing these concerns is essential to reducing weight stigma in healthcare and protecting children from harmful experiences.

## HIGHLIGHTS

- Offers novel insights into parents’ experiences with HCP in weight discussions
- Reveals how parents approach involving children in weight-related conversations
- Shows that parents’ experiences of HCPs range from supportive to stigmatizing
- Uncovers conflicting parental views on including children in weight discussions
- Stresses the need for HCPs to adopt a sensitive and non-judgmental approach

## Introduction

Interactions with healthcare professionals (HCPs) are key to parents’ engagement in childhood obesity treatment [1]. However, parents’ experiences of communication with HCPs during such treatment remain understudied, and only one study has explored parents’ preferences regarding their child’s presence during weight-related clinical visits [2]. In Sweden, where this study was conducted, the prevalence of overweight and obesity among 4-year-old children is slightly above 11%, with significantly higher rates among children living in poverty and in single-parent households [3]. Overweight and obesity in children are typically screened for in routine health assessments at child health care centers, using age- and sex-specific growth charts based on International Obesity Task Force (IOTF) cut-offs [4]. If overweight is detected, children are offered additional health check-ups at the child health care center, including monitoring growth and providing lifestyle advice [5]. Children who meet criteria for obesity are, in principle, referred to outpatient pediatric clinics for further evaluation and treatment. The treatment for childhood obesity is structured around family-centered support, including dietary and physical activity advice, by a multidisciplinary team consisting of pediatricians, nurses, dietitians, physiotherapists and psychologists [6]. However, although obesity can and should be identified from the age of two, it remains relatively uncommon for children aged 2–6 years to be referred for further evaluation or treatment. Given the reliance of childhood obesity treatment on family engagement, the interaction between families and healthcare professionals plays a central role in its effectiveness.

Existing research conducted with parents of children with obesity across various age groups has highlighted problematic aspects of healthcare interactions around childhood obesity. For example, parents of children aged 4–15 years have reported that their concerns about their child’s weight were downplayed by HCPs [7]. Similarly, parents of children aged 5–10 years have reported avoiding health care services due to fear of being blamed for their children’s weight [8]. Concerns about experiencing weight stigmatization have also been documented among parents of children ranging from preschool age up to 18 years [9,10]. Other studies found that parents wanted to shield their preschool-aged children from weight-related discussions due to concerns about potential negative outcomes, such as developing a negative body image or disordered eating behaviors [11,12].

Theoretical perspectives on healthcare interactions and children’s role in Swedish obesity treatment

Healthcare interactions are shaped by both explicit and implicit communication processes, encompassing verbal exchange (e.g. information sharing) and non-verbal cues such as body language, eye contact, and tone [13]. Beyond these observable elements, interactions convey relational qualities including kindness, respect, helpfulness, and perceived human worth [14]. From a theoretical perspective, patient-centered communication fosters trust, enhances engagement, and leads to better health outcomes [15], which is particularly crucial in managing stigmatized conditions like obesity [16]. Best practices in childhood obesity treatment emphasize building a therapeutic alliance with parents through open dialogue characterized by mutual respect and trust [17]. The communicative dynamics between health care professionals, parents, and children in obesity are shaped by structural and normative frameworks [18], yet a gap persists between best-practice recommendations and real-world clinical interactions.

In Sweden, it is standard practice for children to be present in healthcare visits concerning them. This aligns with the Convention on the Rights of the Child, incorporated into Swedish law in 2020, which asserts that children capable of forming their own views have the right to receive age-appropriate information about their health [19]. While this principle guides clinical care, its application in weight-related discussions remains complex as it is unclear whether involving children in conversations about weight as part of growth and development serves the child’s best interests. Furthermore, there is a lack of research examining parents’ perspectives on this matter – our present paper addresses this gap.

## Aim and research questions

The overall aim was to explore how parents of young children (aged 2–6 years) with overweight or obesity experience healthcare interactions during childhood overweight and obesity treatment. Two research questions were addressed:

1. How do parents experience interactions with HCPs during visits regarding their child’s weight?
2. What are parents’ approaches towards their child being present in the room when their weight is being discussed?

## Materials and methodology

### The more and Less Europe study

In this qualitative study, we included parents participating in the More and Less (ML) Europe study which was part of a work package within the EU initiative Science and Technology in children’s Obesity Policy (STOP). The ML Europe study was a randomized controlled trial testing a parent support program as treatment for children aged 2–6 years old with overweight or obesity [20]. It was conducted during 2018–2022 in three European countries: Sweden, Romania, and Spain. This study is based on interviews with Swedish parents.

Children with overweight or obesity according to international cut-offs [4] were referred to the study by family physicians, pediatricians, primary child health care centers, hospitals, or through self-referrals. Following referral, participants were randomized to either the intervention group or the control group. The intervention group received the ML parent support program [21–24] consisting of group sessions focusing on parenting practices to support healthy behaviors, followed by a mobile-based lifestyle support program, the MINISTOP app [22,25]. The control group was referred to standard care, which, in Sweden, was provided at outpatient pediatric clinics. While this care is usually offered only to children with obesity, in the context of the ML Europe study, both children with overweight and obesity were referred to such clinics. At these clinics, parents and their children met with a pediatrician for annual health check-ups with follow-up visits to a pediatric nurse. During the visits, families were offered lifestyle counselling, including advice on food intake, eating behaviors, and physical activity, and monitoring of the child’s weight development. Families were further referred to dietitians and physiotherapists in primary health care if additional support was deemed necessary. As we were interested in capturing parents’ experiences of interactions with HCPs within the structured treatment context provided at these clinics, only participants who received the standard treatment at outpatient pediatric clinics in Sweden were invited to partake in interviews.

### Recruitment and participants

All parents randomized to the control group (*n*=54) were contacted via telephone and invited to partake in individual, semi-structured interviews. Of these, 45 agreed to participate; a majority were female (71%), had a university degree (60%) and were born outside of Sweden (51%). An overview of participant characteristics is presented in Table 1. We were unable to reach seven of the parents and two declined due to time constraints. The interviews were carried out between May and November 2023, about three to four years after treatment initiation.

**Table 1.** Characteristics of the interviewed parents and their children.

**Child**	
*Sex*	n (%)
Male	11 (24)
Female	34 (76)
	Mean (SD)
Age at time of treatment (years)	4.6 (0.96)
Age at time of interviews (years)	7.1 (0.72)
Mean BMI SDS at baseline	2.6 (1.3)
BMI SDS mean change 9 months post-treatment	−0.11 (0.36)
**Parent**	
*Sex*	n (%)
Male	13 (29)
Female	32 (71)
Age (years)	Mean (SD) 40.5 (4.7)
*Country of birth*	n (%)
Born in Sweden	22 (49)
Born outside Sweden	23 (51)
*Education level*	n (%)
University	27 (60)
No university	18 (40)
*BMI*	n (%)
18 < 25	11 (24)
25 < 30	23 (51)
≥30	11 (24)

SD: standard deviation; BMI: Body mass index; SDS: standard deviation score. BMI classifications were determined according to the World Health Organization’s cut-off criteria for BMI [23].

### Data collection

The interviews were carried out via telephone by one of two female researchers: a professor (PN, *n* = 38) and a pediatric nurse and research assistant holding a master’s degree (KN, *n* = 7). PN was the principal investigator of the trial but had no established contact with the participants prior to the interviews. KN worked with data collection in the trial and had met all participating parents and their children. While the interviews were individual, in a few cases, the child was present in the background.

The interviews followed an interview guide developed through discussions within the research team (Appendix A). The questions addressed how parents experienced the standard treatment provided, as well as their perceptions of their interactions with HCPs. Furthermore, the questions addressed parents’ attitudes toward, and experiences of, their children’s presence in healthcare interactions where the child’s weight was discussed. Based on the responses, follow-up questions were posed. The interview guide was refined during the data collection period, with some follow-up questions added.

The interviews lasted 12–46 minutes (28 minutes on average), were audio-recorded and transcribed verbatim by four undergraduate students (*n* = 30) and one doctoral student (MS, *n* = 15).

### Data analysis

MS conducted a reflexive thematic analysis as described by [27] guidance. While this method is commonly applied within interpretivist or constructionist paradigms, we conducted the analysis from a realist epistemological position, consistent with our previous work [12]. This position assumes that participants’ accounts reflect their lived experiences and perspectives on healthcare interactions, which we sought to interpret through a systematic and reflexive engagement with the data.

MS was previously involved in the ML Europe trial and had met some of the parents and their children at the study inclusion stage. Initially, MS read and re-read the transcripts and key segments were extracted into an Excel spreadsheet. Subsequently, MS systematically coded key features of the transcripts relevant to the research questions. When the initial step was finished, codes were examined and grouped based on potential themes and subthemes in an iterative process. This included continuously refining the themes for coherence and distinctiveness. MS met regularly with professor PN and associate professor NN to discuss potential themes and subthemes. In addition, the identified themes were shared twice with all co-authors. The broader team’s feedback was incorporated to refine the themes further until consensus was reached.

### Reflexivity and rigor

This study benefited from the varied experiences among the authors. MS is a doctoral student specializing in childhood obesity stigma. PN, NN, AE, KJ, XRS, and KN are all experienced senior researchers in childhood obesity, obesity stigma, and qualitative methods. In addition, PN served as the principal investigator of the More and Less trial, while AE, KN, and MS were members of the RCT research team. We acknowledge that these experiences influenced the research questions, data collection, interpretation and conclusions drawn.

In addition to the broad expertise among the authors, methodological rigor was promoted through using semi-structured interviews that generated rich and in-depth data. This approach ensured consistency across interviews through the same set of main questions, while also allowing for reflexivity through tailored follow-up questions based on the responses. Furthermore, a majority of the parents in the control group were interviewed, such that a broad range of perspectives and experiences was captured. This was further reflected in the thematic analysis, where both major and minor themes were identified, representing the diversity of parental experiences as recommended by [28].

### Ethical considerations

The trial was approved by the regional ethical board in Stockholm, Sweden (registration number: 2018/2082–31/1). Each participant received both verbal and written information about the study, was given the opportunity to ask questions and provided signed informed consent before taking part in the interviews. Interview transcripts were pseudonymized to ensure participant confidentiality, meaning that each participant was given a study ID and the names of individuals, institutions, and places were removed.

## Results

**Figure 1.**
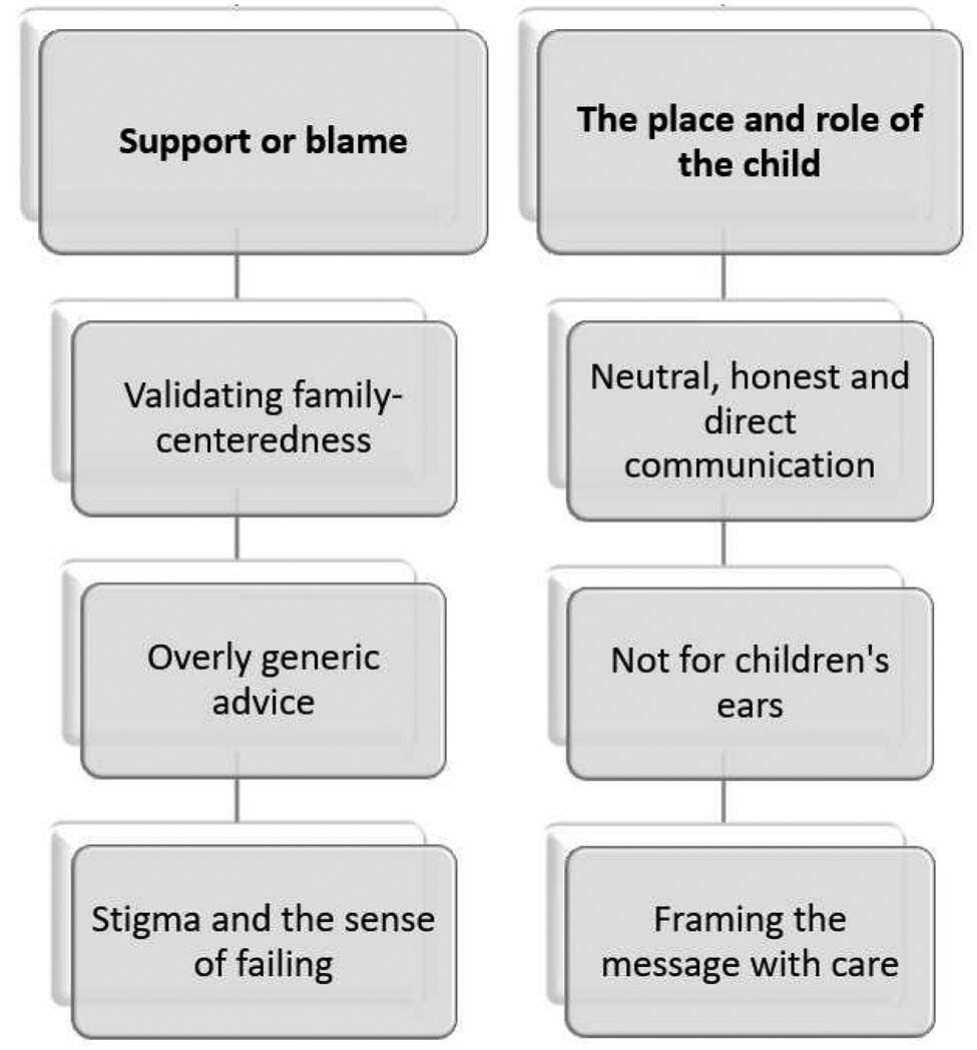
Identified themes and subthemes.

Two main themes, with three subthemes each, were developed; the themes and subthemes are presented in Figure 1 and described in detail below. Quotes from the parents are provided to support each theme, followed by a randomly assigned identification code, the parent’s gender (mother or father), and their child’s age at the start of treatment.

### Support or blame

This theme captures parents’ experiences of interacting with healthcare professionals during their child’s treatment. It illustrates that parents appreciated a collaborative and empathic approach, whereas oversimplified or stigmatizing encounters led to feelings of disappointment, anger, and shame. The theme includes three subthemes: *Validating family-centeredness, Overly generic advice,* and *Stigma and the sense of failing*, each described in detailed below.

#### Validating family-centeredness

When HCPs demonstrated commitment and understanding of the family’s unique situation, parents experienced this positively. According to these parents, successful meetings were characterized by open-ended questions, a respectful approach, and support that aligned with the family’s needs. One father, who was satisfied with his interaction with the HCP, felt that his concerns were met with understanding:

I felt that it [the treatment] met my expectations. I think we were treated well and taken seriously, and it felt like something we were doing together (code 1045, father of a 3-year-old boy).

The quote suggests that the HCP created a partnership with the parent and adopted a constructive, non-judgmental communication around the child’s weight. When the HCP adopted this approach, parents felt empowered to voice their concerns openly and express their needs. This resulted in parents leaving the visit feeling heard, validated, and motivated to engage in their child’s weight management.

#### Overly generic advice

While most parents generally reported positive experiences of healthcare interactions, several parents expressed disappointment. As one father put it: ‘When you use the word treatment, I believe it should include some form of actual intervention’ (code 1089, father of a 5-year-old boy). These feelings often arose because the support provided consisted of generic or repetitive lifestyle advice that parents were already familiar with. Hence, they felt as if the HCP made assumptions about their lifestyle, causing them to feel judged, frustrated, or even patronized:

Sometimes it feels like you’re being patronized … it’s the same talk over and over again. … It’s like telling an adult, ‘You’re overweight, you should eat small portions, watch your calories, and exercise every day.’ Yeah, yeah, everyone knows that. If it were that easy, I wouldn’t be sitting here (code 1116, mother of a 5-year-old girl)

Several parents shared similar feelings and stated that as a consequence, the support provided was ineffective in helping them manage their child’s weight. Additionally, some parents perceived the HCPs as being at a loss when addressing the child’s overweight which left them unable to offer any helpful support. In other cases, parents felt that their concerns were dismissed:

At so many of these visits, he [the HCP] kept saying there’s no problem. …. ‘This will sort itself out with time. There’s nothing wrong’. But at the same time, I can clearly see that he [the child] is bigger than many other kids and not as active. … So, for our family, it’s definitely a problem (code 1125, mother of a 3-year-old boy)

The quote highlights a tension between the professional’s advice and the mother’s weight management expectations.

#### Stigma and the sense of failing

While some parents described HCPs’ support as insufficient or dismissive, others reported more direct forms of stigmatizing practices and interactions. In such cases, the approach went beyond implicit assumptions about the family’s lifestyle to more overt blame and judgement. For example, some parents recalled instances where they felt poorly treated, blamed, or stigmatized by the HCP. Such stigma was often expressed through an unfriendly attitude and insensitive comments about the child’s appearance or the family’s lifestyle habits. One mother of a 5-year-old girl (code 1062) shared a comment made by an HCP: ‘Well, you don’t get fat just from eating vegetables’.

In most cases the participants described, these stigmatizing comments were made in front of the child, making the parents concerned about the child’s self-image and well-being. Some parents noted that their child became visibly affected by it. For example, one mother recalled a visit where her daughter received a comment about her weight. She perceived that this made her daughter more self-conscious about her appearance, leading her to avoid follow-up appointments: “She was with her dad; I was at work. When I came home, she was completely silent. Then she said to me, ‘Mom, they said I was fat. She said I had a big belly’” (code 1038, mother of a 6-year-old girl).

Moreover, a few parents said they felt criticized and as if they had not been attentive enough to their child’s weight changes. For example, one mother, who became upset during the interview, recalled one such experience: ‘She [the HCP] said ‘But you can see that she has a belly, right?’ I mean yes, of course I see, but she’s only three and a half years old!’ (code 1129, mother of a 3-year-old girl). This mother felt that the HCP held her accountable for her child’s weight changes, suggesting that she had failed to fulfill her parental responsibility. Similar descriptions were shared by other parents who felt the HCP insinuated they had not done enough to manage their child’s weight.

Furthermore, some parents articulated internalized feelings of shame and self-blame because of their child’s weight. These parents often felt personally responsible for the situation and believed it was up to them to resolve it. As one mother expressed: ‘It’s entirely my fault too, since she only lives with me. That’s just the way it is. … I don’t really know how to handle it differently, either’ (code 1048, mother of a 5-year-old girl). This sense of ‘failing’ led some parents to feel lonely, isolated, and as though no one could help them.

### The place and role of the child

This theme encompasses parental perspectives on their child’s presence during weight-related conversations in healthcare settings. It illustrates how parents approaches to preparing children for visits ranged from neutral to direct explanations, and how they held contrasting views on children’s presence during weight-related discussions. The theme comprises the subthemes *Neutral, honest, and direct communication, Not for children’s ears,* and *Framing the message with care.*

#### Neutral, honest, and direct communication

When preparing the child for the healthcare visit, parents adopted various approaches. As the children were very young when the treatment started (some were three years old), some parents felt it was unnecessary to provide them with a thorough explanation about the visit’s aim. They simply explained that they were going to a ‘routine check-up,’ to ‘the doctor,’ or, as one mother said, ‘the food lady.’

Some of these were parents of the youngest children, who explained that as their child was so young, they did not fully understand the discussion. Consequently, they were less concerned about the potential risks of their child being present during the conversation. For example, one mother, who also had an older son undergoing obesity treatment, reflected on the age difference: ‘He [her youngest son] was so little … But my [older] son, the one who’s two years older. He goes to [another clinic], as I mentioned, and that can be a bit sensitive for him’ (code 1029, mother of a three-year-old boy). Thus, this mother expressed greater concern for her older son, as his age allowed him to understand more than her younger son, making weight-related healthcare visits more sensitive.

However, some parents, all born abroad, adopted a more direct approach. They openly told their children, who were 5 or 6 years old at the time of treatment, that the clinical visits were intended to help them lose weight, as exemplified by this mother:

I explained to [the child] that we’re going to this visit to manage her weight. They’re going to give us information about how many meals she should eat, what kind of milk she should drink, and what activities she should do (code 1070, mother of a 5-year-old girl).

These parents emphasized that children must be informed about their weight, so they can learn and take personal responsibility for their health. They expressed beliefs that children are more likely to listen if the information comes from a ‘doctor,’ rather than from parents. Furthermore, they argued that addressing the issue openly is more honest than discussing it behind the child’s back. As one father put it: ‘[S]he needs to be there to listen and learn. It’s important. It has to come from a doctor—it can’t always be from mom and dad. That doesn’t work, because she doesn’t always listen then’ (code 1072, father of a 5-year-old girl).

#### Not for children’s ears

In most cases, the clinic required children to attend all weight management consultations, despite some parents expressing a strong preference for not having their child present. These parents often mentioned feeling uncomfortable addressing weight-related issues in front of their child as they feared it could negatively impact their body image and lead to eating disorders. To keep the child unaware of the discussion, some parents preferred that HCPs talk around the issue, avoid explicit mentioning of weight and instead discuss the growth chart. Some parents observed that their child was already aware of their overweight, as illustrated by this mother’s account:

I find it very difficult to talk about food and weight when he’s in the room. I know he understands some of what we’re saying, and he looks at himself and says things like, ‘My big belly.’ It really cuts straight through the heart to hear that. (code 1125, mother of a 3-year-old boy)

Several parents argued that the child’s weight management was primarily a parental responsibility, making the child’s presence unnecessary. They felt that a conversation without the child would have allowed them to speak about weight more openly, as this father expressed:

It gets a bit difficult for us to sit and talk with the doctor who says, ‘Well, do you have any questions? Do you have any concerns?’ Yes, I do, I have many, but I can’t discuss them with you right now while my child is sitting there (code 1052, father of a 3-year-old girl)

Some parents also noted that HCPs felt uncomfortable discussing the child’s weight in front of the child. For example, they recalled examples where the healthcare provider started talking around the subject, spoke English, or asked the child to play in the waiting room in the meantime to prevent them from overhearing the conversation. Despite this, very few parents were offered an appointment without the child.

#### Framing the message with care

Some parents agreed it was appropriate for the child to be present during visits but stressed that both content and how the provider communicated with the child were crucial. For example, several parents preferred to avoid weight-focused messages and instead encourage positive aspects and behavioral progress. In addition, parents emphasized that it is vital to engage with the child at their level, using age-appropriate language and avoiding stigmatizing terms.

As a parent, you don’t want the children to be sitting there while someone talks over their heads about their appearance or… the focus has to be correct. It is incredibly important how one expresses oneself during such visits to avoid planting seeds (code 1049, mother of a 4-year-old girl)

The mother’s reference to ‘planting seeds’ indicates a fear that inappropriate discussions could have lasting and harmful effects on the child’s self-esteem or body image. At the same time, some parents felt that involving the child in discussions about weight is crucial because food and eating are central to their family’s life – ‘everything revolves around food’ (code 1116, mother of a 5-year-old girl). Rather than focus on weight, these parents explained the aim of the visit in terms of health benefits, describing how the visit would help them eat a healthier diet, improve their overall well-being, or prevent future health problems.

We don’t want to say that you’re starting to get fat or anything like that. We haven’t said that. It’s more like… Well, ‘We’re going to check and think about what we’re eating and not eat so much ice cream because it’s not good for our health’ (code 1126, father of a 4-year-old girl)

Parents noted that such an approach allowed them to avoid unnecessary focus on weight and instead fostered a healthy and supportive environment for their child.

## Discussion

In this interview study, we explored how parents of young children experience weight-related conversations with HCPs during treatment. We also explored how parents explain weight-related healthcare visits to their children, and how they feel about their children being present during weight management discussions. We found that several parents had positive and supportive healthcare experiences, while others described feeling poorly treated or unfairly judged by the HCP, including weight stigma directed at the child. This could result in feelings of self-blame for ‘failing’ to manage their child’s weight. Some expressed a preference for having weight management consultations without their child present due to fear of potential unintended consequences for the child, while others perceived that the child’s presence enabled an honest relationship and meaningful lifestyle changes. Several parents stressed the importance for HCPs to adopt a respectful and non-stigmatizing approach.

Our study offers novel insights into parents’ perceptions regarding their young child’s presence during weight management healthcare visits. To date, only one qualitative study involving preschool children has explored parents’ experiences on this matter [2]. In line with our findings, that study showed that several parents preferred to exclude the child from these discussions to avoid potential harm [2]. Similarly, other qualitative studies involving children of various ages, have highlighted the internal conflict parents experience between addressing the child’s weight and fearing negative outcomes [11,12,29,30].

In contrast, a Swedish study found that parents viewed their 4-year-old child’s presence in weight-related discussions as uncomplicated [23]. This rather neutral view was shared by some parents in our study, and some went even further to suggest that having the child present could help them manage their own weight by allowing the child to receive information directly from the HCP instead of the parents. This perspective may reflect underlying weight bias, as it places the responsibility for weight management on the child and emphasizes weight loss over improvements in health, health behaviors, and wellbeing (as indicated by evidence-based clinical practice guidelines) [24]. A recent literature review [31] found that parents of very young children (aged 1-3 years old) often hold negative attitudes towards overweight and obesity (i.e. weight bias). This is concerning, as children depend on their parents for support in weight management, while the focus should be on prioritizing health and well-being rather than weight or appearance. To support parents in this role, they need to be able to voice their concerns and receive support without the presence of their child. This may provide a space for open discussions while minimizing potential harm to the child’s body image. Parents may also need education about obesity as a chronic disease so they can better understand that the outcomes of obesity treatment programs go beyond weight loss, including improvements in health and psychological wellbeing and the prevention of obesity-related comorbidities.

Additionally, when children are present, it is crucial to consider the content, context, and the child’s developmental stage, ensuring that the provided information is tailored to their age and free of stigmatizing language. The importance of such an approach has been emphasized in previous research [32] and aligns with evidence-based clinical practice guidelines [17]. Despite this, and in line with previous research [33], several parents in our study perceived that tailored support was lacking. This highlights the need to implement clear, practical education for HCPs as well as models of care to guide HCPs in addressing weight management, without restricting their ability to tailor clinical visits to individual families. Furthermore, parents’ differing preferences regarding their child’s involvement and the HCPs’ approach illustrate the complexity HCPs face when working with families of children with obesity. Before initiating treatment, HCPs need to understand and consider the family’s unique circumstances and parents’ expectations of treatment.

Notably, a previous study found that 4-year-old children enjoyed participating in weight management conversations when they were involved in an age-appropriate manner [34]. This suggests that, if the information is tailored to their age, young children can actively participate in such discussions. This may support their understanding and involvement in their own care. However, there remains a need for a greater understanding of when and how children’s presence is appropriate and what implications these consultations may have for them. In addition, further research is needed to explore HCPs’ preferences and skills in addressing this issue.

Moreover, while participants shared experiences of positive healthcare encounters, our study reveals that some parents were subjected to weight stigma, alongside a widespread lack of understanding about the complex factors contributing to overweight and obesity. For instance, parents reported that HCPs made insensitive comments about their child’s weight, which, in several cases, were intended. This confirms previous studies that reported on HCPs’ stigmatizing attitudes toward individuals living with obesity [35], including HCPs working with children [36–38]. Such experiences discouraged parents from attending follow-up visits with their children, which has also been demonstrated in other qualitative studies [2,11]. These findings highlight the importance of awareness among HCPs about the complex factors contributing to overweight and obesity and the harms of weight stigma. To address this, promising approaches include early and continuous education about obesity and weight bias sensitivity training [39]. Additionally, to facilitate constructive consultations around weight, it is crucial for HCPs to seek permission from parents to discuss the child’s weight and to do so sensitively and respectfully [16]. Crucially, as [40] point out, experiences of stigma can lead individuals to internalize negative beliefs about themselves (see also 41]. Such internalization was evident in our study, where parents expressed feeling responsible for their child’s excess weight. Similar findings have been reported in other qualitative studies, which show that parenting a child with overweight or obesity is closely tied to feelings of shame and guilt [29,42,43]. It is important to recognize, however, that obesity is a chronic disease, and as such, blaming parents will likely do more harm than good.

### Strengths and limitations

A key strength of this study is the size and composition of the sample. Of all the parents in the original sample, we interviewed a substantial proportion (83%), which enabled us to capture a diverse range of perspectives across the group. Almost one-third of our sample were fathers, which we consider an additional strength as fathers’ perspectives are lacking in childhood obesity research [33]. However, while the study provides novel insights into parents’ views on children’s presence in obesity treatment consultations, it does not explore the extent to which these views impacted on parents’ continued engagement with treatment and long-term weight management. Furthermore, parents’ education levels may have influenced their experiences of interacting with HCPs. However, we did not consider this factor in our analysis, which we acknowledge as a limitation. Lastly, since the interviews were conducted several years after treatment initiation, responses may have been subject to recall bias.

## Conclusion

Taken together, parents have different experiences of discussing their young children’s weight with HCPs, ranging from empathic interactions, where HCPs validate parents and frame the weight talk sensitively, to negative interactions, where HCPs blame parents and reproduce weight stigma. Furthermore, whether or not parents want their child to be present in weight-related discussions varies, with some feeling that these conversations are inappropriate for children and others considering them valuable learning experiences. Given this finding, HCPs should adopt a sensitive and non-judgmental approach when discussing children’s weight with parents, and tailor communication to both parents and children. By doing so, HCPs can help mitigate the burden of stigma and create an open, constructive dialogue during weight-related clinical visits. To support this approach, we suggest that healthcare systems implement continuous education and structured care models.

## Data Availability

The transcripts/data from this qualitative study are not accessible due to confidentiality agreements made with the participants.
